# Unilateral hearing loss: benefits and satisfaction from the use of hearing aids

**DOI:** 10.1590/S1808-86942011000200012

**Published:** 2015-10-19

**Authors:** Maria Renata José, Patrícia Danieli Campos, Maria Fernanda Capoani Garcia Mondelli

**Affiliations:** 1 Graduanda do 4° ano do Curso de Fonoaudiologia da Faculdade de Odontologia de Bauru, Universidade de São Paulo. Discente; 2 Mestranda do Programa de Pós Graduação em Fonoaudiologia da Faculdade de Odontologia de Bauru, Universidade de São Paulo. Fonoaudióloga da Clínica de Fonoaudiologia da Faculdade de Odontologia de Bauru, Universidade de São Paulo; 3 Doutora em Distúrbios da Comunicação Professora Doutora do Curso de Fonoaudiologia da Faculdade de Odontologia de Bauru, Universidade de São Paulo. Trabalho realizado na Clínica de Fonoaudiologia da Faculdade de Odontologia de Bauru, Universidade de São Paulo

**Keywords:** hearing aids, hearing loss, unilateral, questionnaires

## Abstract

Aunilateral hearing loss is characterized by reduced hearing in one ear. The problems caused by sensory deprivation can be minimized with the use of hearing aids (HA).

**Aim:**

To analyze the correlation between the prescribed grain and the insertion gain difference and with the results obtained regarding the benefit and satisfaction with the use of hearing aids in unilateral hearing impaired patients.

**Materials and Methods:**

Prospective study with 15 subjects, mean age of 41.6 years, of both genders, users of hearing aids effectively. We used the International Questionnaire Results for hearing aids (International Outcome Inventory for Hearing Aids - IOI-HA), measured with a probe microphone.

**Results:**

The mean values in the analyses of the IOI-HA per item were positive and higher than four points. In relation to the objective measures, the frequencies in which we obtained the gain values which were closer to the target were: 1K Hz, 2K Hz and 500 Hz, respectively.

**Conclusion:**

The satisfaction of individuals using hearing aid unilaterally is not completely correlated to the prescribed gain, because even if the target is not being reached in some frequencies, the individuals were pleased as to the use of their hearing aids.

## INTRODUCTION

Hearing is one of the fundamental senses in life, playing a very important role in society, being considered the basis for human communication development. Individuals with hearing impairment may suffer handicaps in their social, psychological and professional lives^1^.

According to the 2002 Census (IBGE)^2^, 5.7 million Brazilians stated they had hearing impairment (HI). This number is probably much higher, because often times the problem is not perceived, or it is denied by the individuals. The non-acceptance does not lead to treatment, and this may worsen the frustration of not being able to hear and the individual isolates himself.

Many are the causes which contribute to the increase in the number of hearing impaired individuals: presbycusis, hereditary diseases, metabolic disorders, use of ototoxic drugs, acoustic trauma, excess noise, different types of neoplasia, infections and vascular damage. Among the resulting effects, we stress anxiety, frustration, feeling of insecurity, emotional instability, depression, social phobia, a feeling of frustration and incapacity to guide oneself^3^.

Unilateral hearing loss is characterized by hearing reduction in only one ear and it happens, predominantly among males^4^. In one study^5^ they found the main etiologies to be mumps, ototoxicity, meningitis, NIHL, German measles, head injury and sensorineural unilateral hearing loss of unknown cause.

The effects of unilateral hearing loss are lower than the ones caused by bilateral hearing loss, which can also cause problems. Under room noise, individuals with unilateral hearing loss find more difficulties than their normal hearing counterparts to understand speech, even when the best ear is positioned towards the source of speech. Moreover, the spatial location of the sound source is compromised^6^.

The problems brought about by sensorial deprivation can be minimized with the use of an Individual Sound Amplification Device (ISAD) - hearing aid, which enables one to bring back the perception of speech sounds, besides environmental sounds, bringing about an improvement in communication skills^1^.

There are many factors which contribute to the successful use of amplification. Age, type and degree of the hearing loss, physical factors (ear size and manual dexterity), auditory processing skills, prior use of sound amplification device and hearing loss extension, which together, have a crucial role for accepting the amplification. Added to this, the perception of the auditory handicap, cost, personal expectations, satisfaction, performance and benefits may indicate whether or not we will have a happy and satisfied user of a sound amplification device^7^.

Acceptance may be characterized in two ways: either the device is accepted or rejected; but it can also be characterized as a psychological process of dealing with the idea and the sensation of sound amplification, at the same time as it incorporates the device to your lifestyle. Satisfaction is built according to the subjective impressions of the individual. Thus, it becomes clear that, while there is no acceptance, there will never be satisfaction, just like not all acceptance and benefit associated with the device are enough parameters to guarantee satisfaction. While the benefits can be shown through objective tests, personal satisfaction is a very personal assessment of the value of the sound amplification device after a given time of use^8^.

It is possible to state that the checking procedures, as the functional gain and the insertion measures, are not enough to assess the individual's satisfaction with the device in daily communication tasks. There was a growing interest in the development of validation procedures which enabled to study the user's benefit outside the clinical settings, making up self-assessment questionnaires^9^.

The self-assessment is a simple, fast and efficient procedure which enables the assessment of the individual in his process of ISAD's adaptation. This procedure enables the comparison between different devices and/ or calibrations, as well as the assessment of the benefit achieved with the same ISAD along time, enabling the user to recognize the advantages provided by the ISAD in relation to the individual's auditory difficulties and psychosocial disadvantages. Thus, by means of questionnaires which enable the measuring and analysis of these auditory difficulties or that of the *handicap*, it is possible to optimize the time it takes for the person to adapt him/ herself to the amplification^10^.

There are numerous assessment instruments which have scores used to assess the level of individual satisfaction, even because there are numerous factors which influence different dimensions associated with the use of a sound amplification device^8^.

In Brazil, some self-assessment questionnaires, the APHAB (*Abbreviated Profile of Hearing Aid Benefit*), the IOI-HA (*International Outcome Inventory for Hearing Aids*), the HHIE (*Hearing Inventory for the Elderly*) and the HHIA (*Hearing Handicap Inventory for the Adults*) among them, were translated and adapted to our reality in Brazil, investigating the degree of user satisfaction, the benefits obtained from using ISADs and the reduction in auditory capacity with the use of amplification, and others with the goal of comparing the benefit of different Technologies and checking the ISAD fitting by means of objective and subjective measures^9^.

The benefit has been traditionally assessed by means of objective data, in other words, the hearing loss nature and severity are defined as basis in the assessment of the thresholds obtained by means of equipment calibration in controlled environments. Thus, the hearing performance improvement enjoyed by individuals with ISADs can be obtained by means of measurements such as the insertion gain which is understood as a benefit^9^.

Because of the scarcity of studies concerning unilateral hearing loss, the goal of this study is to objectively and subjectively measure the benefit and the satisfaction of the individuals using unilateral ISADs.

## MATERIALS AND METHODS

Patient selection and assessment started after the Ethics Committee approved the study under process # 001/2009 and signed the Free and Informed Consent Form.

This cross-sectional contemporary cohort study was carried out with 15 individuals with a mean age of 41.6 years, of both genders, (12 females and 3 males).

Participant inclusion criteria were:

- Age range: adult individuals (18 to 60 years);
- Hearing loss: mixed unilateral or sensorineural of moderate, severe and profound levels;
- Effective ISAD user for more than six (6) months.

The HI degree was classified using the audiometric thresholds of the 500; 1,000; 2,000; 3,000 and 4,000 Hz frequencies: mild HI (mean between 26 and 40 dBHL), moderate HI (mean between 41 and 60 dBHL), severe HI (mean between 61 and 80 dBHL) and profound HI (mean higher than 81 dBHL), according with the WHO^11^.

We used the *International Outcome Inventory for Hearing Aids* - IOI-HA developed as the product of an international workshop on self-assessment measures in auditory rehabilitation^12^, ^13^. Currently, the IOI-HA questionnaire is included in the Hearing Aid Selection and Fitting Form (Ordinance SAS/MS # 587, of 10/07/2004)^14^. This questionnaire assesses seven domains deemed important for the successful use of a hearing aid (use, benefit, limitations in residual activity, satisfaction, impact on others and quality of life) (Attachment 1).

The questionnaire was used as an individual interview in order to make sure all the questions would be answered and that the individual had fully understood the question.

During the checking process, we carried out measures with a probe microphone according to indications from international protocols^15^. Thus, the data was collected using the Unity (Siemens) equipment, following the criteria below:

- we inserted the threshold tonal audiometry data, obtained through air conduction and bone conduction in the *software* used for the probe microphone measures in a way as to generate the gain prescribed by the NAL-NL1 rule.
- with the aim of making the probe tube acoustically transparent we did the calibration, positioning the tube in the horizontal plane at 30cm away from the speaker and near the reference microphone.
- the patient was seated at 50cm away from the sound speaker with the ears in a horizontal plane vis-à-vis the speaker and at 1.5m away from the walls of the room and at a 0^º^ azimuth in relation to the speaker.
- the insertion of the probe microphone in the external acoustic meatus placed at 27-30mm of depth in the external acoustic meatus, using the method in which the probe tube is placed at approximately 3mm from the tip of the ear mold.
- In order to obtain the REIG, the procedure was carried out in the following sequence: measuring response on the external ear without the ISAD *Real Ear Unaided Response* (REUR) followed by measuring the response with the ISAD in the external ear - *Real Ear Aided Response* (REAR) thus obtaining the *Real Ear Insertion Gain* (REIG) using the following calculation: REIG=REAR-REUR. At the end, we noticed that the REIG result reached the target rule, in other words, the calculated target considering the prescription rule formula: NAL-NL1 by previous selection of the equipment software.

Measurements were carried out with stimuli of 50, 65 and 80 dBSPL of the modulated speech type^16^ because it is the stimulus which comes closer to the continuous speech discourse. In order to do the procedures, the extra resources such as feedback control and noise reduction were turned off from the ISAD's programming so as to avoid its influence on the analyzed responses^17^.

We used description by mean and absolute values for the statistical study.

## RESULTS

We scored the IOI-HA instrument of the 15 individuals who answered the questionnaire. On Tables 1 and 2 we can find the answers associated with the daily use, limitations, satisfaction, restrictions, social activities and quality of life.

**Table 1:** Distribution of the answers in each domain of the IOI-HA instrument.

Item	Mean	Standard Deviation
Daily use	4.53	0.83
Benefit	4.00	0.53
Limitations	4.00	1.13
Satisfaction	4.60	0.51
Restrictions	4.73	0.59
Social activities	4.13	1.30
Quality of life	3.53	1.13

Total	29.53	4.07

**Table 2:** Distribution of the answers from each individual concerning the different domains.

Questions							Individuals								
	1	2	3	4	5	6	7	8	9	10	11	12	13	14	15
Daily use	2	5	5	5	5	4	4	5	4	5	5	5	5	4	5
Benefit	3	4	4	4	5	3	4	4	4	4	4	4	4	4	5
Limitations	4	5	4	5	5	1	3	5	5	4	4	3	5	3	4
Satisfaction	4	4	5	5	5	4	4	5	5	5	5	5	4	4	5
Restrictions	5	4	5	5	5	5	5	5	5	5	3	5	5	4	5
Social activities	1	4	5	5	5	3	5	5	5	5	4	3	5	2	5
Quality of life	3	4	4	4	5	4	2	4	3	2	5	4	4	1	4

We created graphs so as to better see the results associated with the insertion gains in the frequencies of 500; 1,000; 2,000; 3,000 and 4000 Hz.

## DISCUSSION

There are numerous protocols available to use in the selection and fitting of a hearing aid, such as the one from Ordinance 587 from the Health Department^14^, *International Society of Audiology*^18^, Valente^19^, Matas & Iório^20^, with different types of procedure; however, they are not unanimous, just like it happened in the present study, there was a need to carry out an objective assessment as well as a subjective one with validated questionnaires and orientation at different stages of the hearing aid selection and fitting.

The application of these procedures in the clinical routine requires knowledge of the ISAD technology, which is to be fit to a patient, and the tests chosen for application^21^, considering the necessary resources, must be selected respecting the particularities of each individual^22^. To check whether or not the hearing aid features were achieved is crucial to the fitting process^18^.

In order to analyze the benefit and satisfaction, we used the IOI-HA questionnaire, and the use of validated questionnaires is a must in a gold standard protocol^23^ with the use of objective and subjective tests geared towards a good communication and proper quality of life for the individual with hearing impairment.

As far as the use is concerned, 66.67% of the individuals reported using the ISAD for more than 8 hours per day, and 26.67% reported uses between 4 and 8 hours per day, and it was possible to notice that all the patients effectively used their hearing aids.

As far as the benefit is concerned, 73.34% reported that the hearing aid helped them much in the situations in which before they had major hearing difficulties; 13.33% of the individuals reported that the ISAD helped them moderately in the situations in which they had hearing difficulties; and 13.33% reported that the hearing aid helped them very much in the situations in which before they had hearing difficulties.

As to the residual limitation in activities, 40% of the individuals reported that they did not have any difficulties in daily hearing situations, 33.33% had little difficulties; and 20% reported still having moderate difficulties in these activities, even with the use of ISAD. These data tell us that the users of unilateral ISAD had improvements concerning the hearing difficulties they had had before the amplification.

When asked about satisfaction concerning the use of the hearing aids, 60% of the individuals reported that it is really worth using the ISAD; and 40% reported that it is reasonably worth using them unilaterally.

Concerning the restriction towards daily activities after fitting the individual sound amplification device, 80% of the individuals reported that with the unilateral fitting of the devices, their hearing difficulties no longer affected their daily activities.

As to the impact the hearing loss causes on people, 60% of the individuals reported that their hearing difficulties did not affect or bother other people; 13.33% reported it affected moderately their relations with other people.

And finally, when the individuals were asked about their quality of lives associated with the use of the sound amplification device, 53.33% reported that after fitting the hearing aid, they became happier with life; 13.33% reported no changes as improvements in quality of life; 13.33% reported a little more happiness in with life after fitting the ISAD; and 13.33% reported a lot more happiness with life after they started using their hearing aids. This data depicts the importance of using these devices in order to enable a better quality of life for those individuals with unilateral hearing loss.

IOI-HA was used for being a brief, encompassing type of measurement, accessible to different cultural and social factors for use and different types of comparisons^12^ in this study focusing on the satisfaction of unilateral sound amplification users. Nonetheless, despite this self-explanatory characteristic of the questionnaire, which does not require additional help to be answered^13^, in this study it was applied by the researcher in charge in order to make sure the individuals understood the questions and answers.

Still concerning the IOI-HA, we may say that the mean values obtained in the analysis per item were positive and higher than 4 points, keeping in mind that the maximum score per question is five. Consequently, the analysis of the summation of all the questions was also positive, indicating a good subjective result in ISAD fitting.

Cox and Alexander^15^ also found a high score in the individuals they assessed in their study using the IOI-HA questionnaire, suggesting favorable attitude concerning their ISAD. They commented on the probable questionnaire sensitiveness to detect individuals with a negative experience regarding the sound amplification.

**Chart 1:**
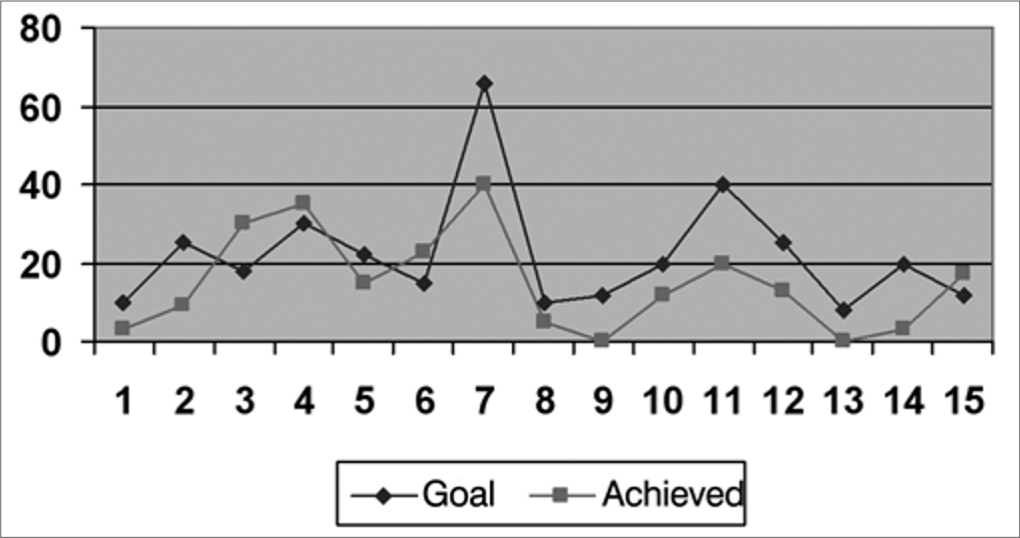
Values of the prescribed gain and the insertion gain at 500 Hz. Target Response Obtained Response

**Chart 2:**
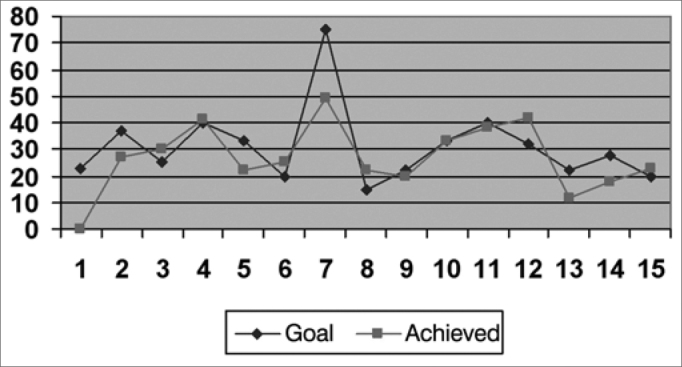
Values of the prescribed gain and the insertion gain at 1000 Hz. - Target Response Obtained Response

**Chart 3:**
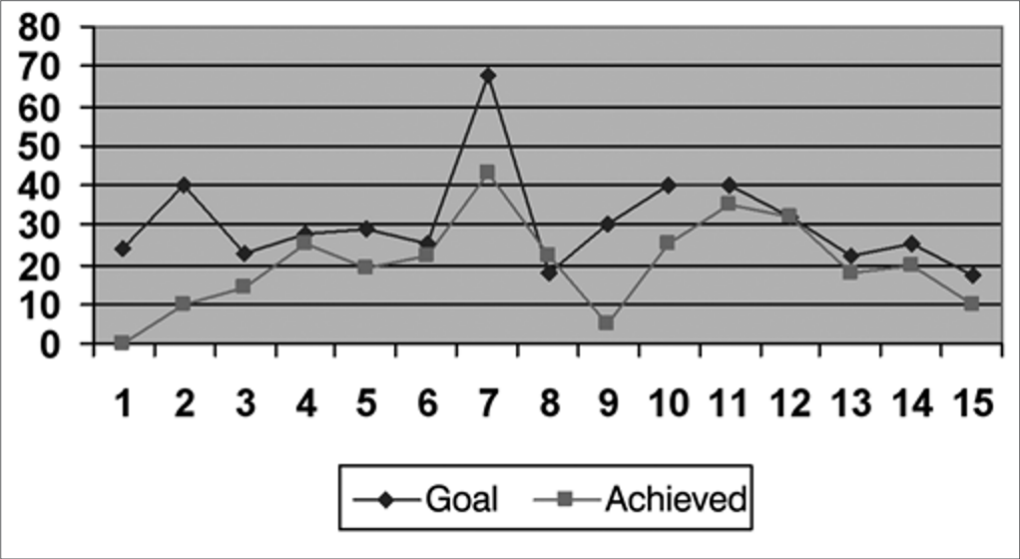
Values of the prescribed gain and the insertion gain at 2000 Hz. - Target Response Obtained Response

**Chart 4:**
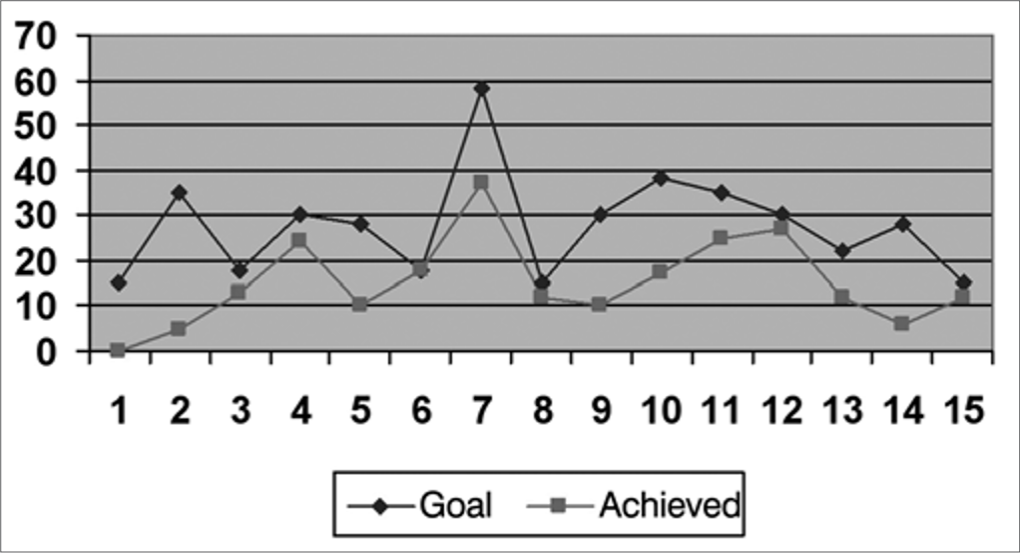
Values of the prescribed gain and the insertion gain at 3000 Hz. - Target Response Obtained Response

**Chart 5:**
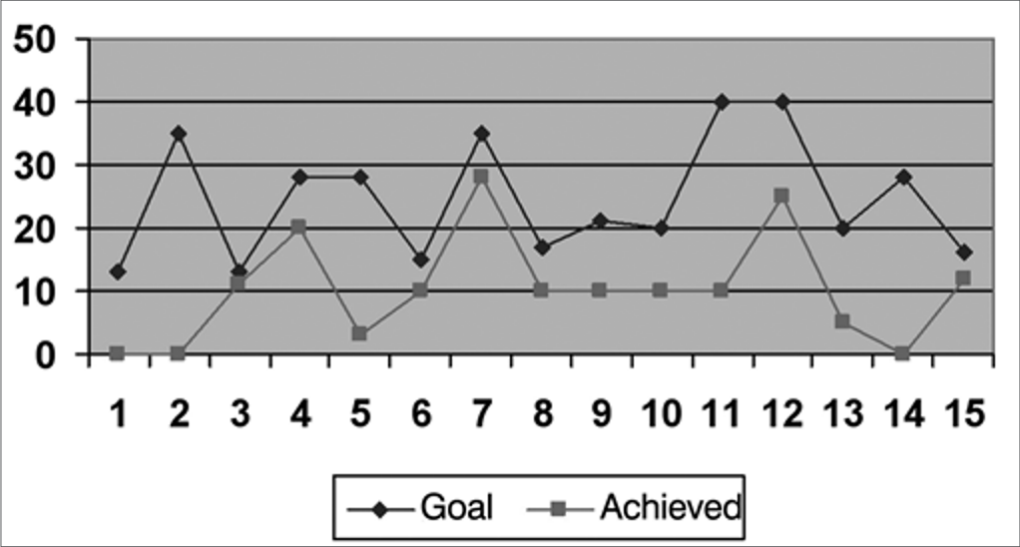
Values of the prescribed gain and the insertion gain at 4000 Hz. - Target Response Obtained Response

As far as the objective measures go, they noticed that the frequencies in which the gain obtained was closer to the target were: 1,000 HZ; 2,000 Hz and 500 HZ, respectively (Charts 2, 3 and 1), and in the frequencies of 3,000 HZ and 4,000 Hz (Charts 4 and 5), in average, half of the individuals did not reach the prescribed value. The only individuals who reached the target in all the frequencies assessed were: 4, 6, 8 and 15, and individuals 4, 8 and 15 had scores higher than 30 in the IOI-HA questionnaire, showing their satisfaction concerning the use of their ISAD, and individual 6 scored 24 (Table 2), showing that even reaching the prescribed gain, he was not entirely pleased with the use of a hearing aid. Contrary to individual 5, who even not reaching the *target* in all the frequencies assessed, had a maximum score in the IOI-HA questionnaire.

We stress that those individuals who were pleased with the use of sound amplification, even not reaching the target, likely because one of the ears did not have normal hearing, since it is known that when the target is not reached, there is a loss for the hearing impaired concerning speech reception and understanding. This indicates that by not doing the checking with objective measures compromises the values programmed in the ISAD, since these can be different from the prescribed target value. In fact, the checking process is crucial, and it is possible to identify the performance of the amplification being provided - both an under-amplification, which may bring about losses in the amplification of speech sounds, as well as an over-amplification, which may cause discomfort and even worsening of the hearing loss^24^.

Speech and hearing therapy requires studies which may contribute to the fitting of a hearing aid in unilateral hearing loss cases, especially concerning the way with which the checking process is carried out and the use of objective measures in the centers certified by the National Policy for Hearing Health Care^14^.

In terms of the insertion gain, its results are extremely versatile, and when well utilized, the method enables the recording of the ISAD performance. This is a powerful tool in the process of selecting and fitting these devices, providing objective data and essential information to the process, enabling greater precision in the adjustments and in the evaluation of the amplification characteristics received by the hearing impaired individual^25^. The insertion gain is little applied vis-à-vis its importance^23^ and, as a consequence, there are but a handful of publications, which is too few to provide for greater discussions, especially associated with unilateral hearing loss.

## CONCLUSION

By means of the self-assessment questionnaire we report on the satisfaction of individuals with unilateral hearing aids, even when the gain necessary to overcome the difficulties brought about by hearing impairment is not reached.

The satisfaction of those individuals users of unilateral hearing aids is not totally associated with the prescribed gain, even though this is an important characteristic for the effective fitting of a hearing aid.

ATTACHMENT **International Outcome Inventory for Hearing Aids (IOI-HA)**

1 Consider the time during which you used your hearing aid in the last two weeks. For how many hours did you use it during a normal day?
  ( ) Did not use it
  ( ) Less than 1 hour per day
  ( ) Between 1 and 4 hours per day
  ( ) Between 4 and 8 hours per day
  ( ) More than 8 hours per day
2 Think about in which situation you would like to be able to hear better before and after obtaining the hearing aid. In the last two weeks, how did the device (s) help you in this same situation?
  ( ) It did not help at all
  ( ) It helped a little
  ( ) It helped moderately
  ( ) It helped much
  ( ) It helped very much
3 Consider again the same situation in which you would like to be able to hear better before and after the ISAD. Which is the level of difficulty you still have in this same situation using the hearing aids?
  ( ) Very much difficulty
  ( ) Much difficulty
  ( ) Moderate difficulty
  ( ) Little difficulty
  ( ) No difficulty at all
4 Taking everything into account, do you think it is worth using hearing aids?
  ( ) It is not worth it
  ( ) It is a little worth it
  ( ) It is moderately worth it
  ( ) It is much worth it
  ( ) It is very much worth it
5 Think about your last week using the ISADs. How much did your hearing problem affected you in your activities?
  ( ) Very much
  ( ) Much
  ( ) Moderately
  ( ) A little
  ( ) Not at all
6 Consider the last two weeks using the ISADs. How much did your hearing problems affect or bothered other people?
  ( ) Very much
  ( ) Much
  ( ) Moderately
  ( ) A little
  ( ) Not at all
7 Taking everything into account, how do you think your ISADs change your joy in living or your enjoyment of life?
  ( ) To worse, or less joy in living
  ( ) There was no change
  ( ) A little more joy in living
  ( ) Much joy in living
  ( ) Very much joy in living
